# The effectiveness of a multidisciplinary intervention strategy for the treatment of symptomatic joint hypermobility in childhood: a randomised, single Centre parallel group trial (The Bendy Study)

**DOI:** 10.1186/s12969-018-0298-x

**Published:** 2019-01-08

**Authors:** Peter Bale, Vicky Easton, Holly Bacon, Emma Jerman, Laura Watts, Garry Barton, Allan Clark, Kate Armon, Alex J. MacGregor

**Affiliations:** 1 Norfolk and Norwich University Hospital NHS Trust, Colney Lane, Norwich, UK; 2 Norfolk Community Health and Care NHS Trust, Norwich, UK; 3 Norwich Medical School, University of East Anglia, Norwich, UK

**Keywords:** Hypermobility, Pain, Children and young people, Physiotherapy, Occupational therapy

## Abstract

**Introduction:**

Joint hypermobility is common in childhood and can be associated with musculoskeletal pain and dysfunction. Current management is delivered by a multidisciplinary team, but evidence of effectiveness is limited. This clinical trial aimed to determine whether a structured multidisciplinary, multisite intervention resulted in improved clinical outcomes compared with standard care.

**Method:**

A prospective randomised, single centre parallel group trial comparing an 8-week individualised multidisciplinary intervention programme (bespoke physiotherapy and occupational therapy in the clinical, home and school environment) with current standard management (advice, information and therapy referral if deemed necessary).

The primary endpoint of the study was between group difference in child reported pain from baseline to 12 months as assessed using the Wong Baker faces pain scale. Secondary endpoints were parent reported pain (100 mm visual analogue scale), parent reported function (child health assessment questionnaire), child reported quality of life (child health utility 9-dimensional assessment), coordination (movement assessment battery for children version 2) and grip strength (handheld dynamometer).

**Results:**

119 children aged 5 to 16 years, with symptomatic hypermobility were randomised to receive an individualised multidisciplinary intervention (I) (*n* = 59) or standard management (S) (*n* = 60). Of these, 105 completed follow up at 12 months. No additional significant benefit could be shown from the intervention compared to standard management. However, there was a statistically significant improvement in child and parent reported pain, coordination and grip strength in both groups. The response was independent of the degree of hypermobility.

**Conclusion:**

This is the first randomised controlled trial to compare a structured multidisciplinary, multisite intervention with standard care in symptomatic childhood hypermobility. For the majority, the provision of education and positive interventions aimed at promoting healthy exercise and self-management was associated with significant benefit without the need for more complex interventions.

**Trial registration:**

The trial was registered prospectively with the national database at the Clinical Research Network (UKCRN Portfolio 9366). The trial was registered retrospectively with ISRCTN (ISRCTN86573140).

**Electronic supplementary material:**

The online version of this article (10.1186/s12969-018-0298-x) contains supplementary material, which is available to authorized users.

## Background

Joint hypermobility is common in childhood, with a high prevalence reported in the literature when adult criteria are applied [1–7]. Hypermobile joints are not synonymous with morbidity; for example, gymnasts, musicians and athletes can use this flexibility to their advantage [8, 9]. However, pain has been reported to occur in as many as 55% of all children with hypermobility [10] and children with symptomatic hypermobility account for a large proportion of referrals to tertiary paediatric rheumatology services [11]. Symptomatic hypermobility (previously referred to as joint hypermobility syndrome (JHS)) is varied in its presentation and is challenging both to diagnose and manage, reflected by a wide variation in practice.

Given the frequency of this presentation there has been a need to develop consistent approaches to treatment. Management guidelines produced by The British Society for Paediatric and Adolescent Rheumatology based on clinical consensus advocate a multidisciplinary approach including physiotherapy (PT), occupational therapy (OT) and podiatry (Additional file 1), with an emphasis on self-management and delivered in clinical, home and school settings. The therapeutic interventions are often targeted at increasing muscle strength and proprioception in the affected joints and limbs [12–15]. However, objective evidence of their effectiveness is almost completely lacking [15].

## Methods

### Objectives

This study aimed to determine the effectiveness of a multidisciplinary care package in children diagnosed with symptomatic hypermobility by comparing a structured, bespoke programme of treatment delivered through a team of clinicians, physiotherapists and occupational therapists in clinical, home and school settings, with standard care.

### Design

Prospective single centre randomised parallel group trial comparing an 8-week individualised multidisciplinary, multisite intervention programme (I) with current standard care (S).

### Setting

Jenny Lind children’s department at the Norfolk and Norwich University Hospital NHS Trust, UK. This is a regional centre for paediatric rheumatology referrals.

### Patients

Children aged 5–16 years old referred to the paediatric rheumatology clinic between October 2010 and November 2012 were eligible for inclusion. Referrals came from community and general paediatrics, orthopaedics, physiotherapy and occupational therapy and general practice.

Patients were recruited between 7th of January 2011 and 14th of December 2012. The final twelve month follow-up assessments were conducted between the 5th of January 2012 and the 20th of December 2013.

### Inclusion criteria

The service accepts referrals up to age 16 years old. In the absence of validated diagnostic criteria for symptomatic childhood hypermobility (or childhood JHS), children who were experiencing pain, functional or coordination problems considered to be secondary to hypermobility by an experienced paediatric rheumatologist were invited to take part, who met the following criteria:

A minimum Beighton score of 4 or more [16], or Bulbena score of 5 or more (males) and 6 or more (females) (see Table 1) [17].

**Table 1:** Classification of hypermobility

Site	Criterion	Beighton	Bulbena
Thumb	Apposition to forearm	x	x
5th Metacarpophalangeal joint	Passive hyperextension > 90 degrees	x	x
Elbow	Hyperextension > 10 degrees	x	x
Knee hyperextension	Hyperextension > 10 degrees	x	
Trunk	Flexion to place hands flat on floor with legs straight	x	
Ankle	Dorsiflexion > 20 degrees		x
Shoulder	External rotation > 85 degrees		x
Hip	Passive abduction > 85 degrees		x
Patella	Passive shift to lateral side of tibia		x
1st Metatarsophalangeal joint	Passive hyperextension > 90 degrees		x
Knee flexion	Heel to contact buttocks		x
Ecchymoses	Presence after minimal trauma		x
	Total point available	9	10
	Score to determine hypermobility	4 out of 9	Males 5/10
			Females 6/10

Musculoskeletal pain in one or more areas of the body for at least 3 months duration.

### Exclusion criteria

Participants were excluded if identified by an experienced consultant paediatric rheumatologist (KA) to have inflammatory joint disease, heritable disorder of connective tissue except Ehlers Danlos hypermobility type, presence of other chronic illness such as chronic pain syndrome or chronic fatigue syndrome using available diagnostic criteria [18–20]. Children under 5 years were excluded, anticipating they would have difficulty complying with the home exercise programme. In addition, children were excluded where, in the clinician’s judgement they were unable to comply with the protocol .

### Baseline assessments

Assessments were carried out by a designated paediatric physiotherapist (VE) assisted by the trial coordinator (LW). Written informed parental consent and patient assent was obtained. Demographic data were collected using a standardised questionnaire.

Specific evaluations included an assessment of:

- **Joint laxity**, clinical evaluation of the extent and distribution of joint laxity by applying the Beighton and Bulbena scoring systems (Table 1).
- **Pain**, using the Wong Baker faces pain scale (0–5) and self-reporting of sites of pain [21]. Children were asked to report their pain over the previous week.
- **Parent reported pain,** using a visual analogue scale (VAS) of 0–100.
- **Physical function,** using the Child Health Assessment Questionnaire (CHAQ) [22].
- **Health related quality of life** (HRQoL) using the 9 dimensional Child Health Utility (CHU9D) [23].
- **Motor skills and coordination**, using the Movement Assessment Battery for Children version 2 (M-ABC2) [24, 25].
- **Grip strength**, using a standardised hand held pneumatic dynamometer (White Plains, New York) taking 3 readings and recording the highest achieved.

### Interventions

All children with confirmed symptomatic hypermobility were offered standard management and given written information regarding the study. Those who showed an interest were contacted by the trial coordinator by telephone after a minimum of 48 h to answer any remaining questions and determine their willingness to take part. The trial coordinator contacted families both by telephone and in writing to achieve maximum retention of participants throughout the study. If no reply was received or if 3 re-scheduled appointments were not attended they were considered lost to follow up.

### Standard care group

Standard care followed usual practice at the hospital and consisted of a single paediatric rheumatology clinic appointment (typically of around 30–40 min) with a Paediatric Rheumatologist (KA) who diagnosed symptomatic hypermobility. All patients and families received verbal information and advice on management including discussing the biomechanical origin of the pain, the need for good muscle strength, examples of appropriate activities and a standard (Arthritis Research UK) information leaflet [26]. Referrals to PT, OT and orthotics were made where deemed clinically necessary.

### Multidisciplinary intervention group

The multidisciplinary intervention was developed and refined by the research team following a systematic review of the literature [27], national consultation with specialist paediatric rheumatology therapy departments and informal consensus. The intervention comprised a programme of 3 individualised physiotherapy sessions addressing problem areas, and promoting improvements in stability and strength; gait analysis and provision of foot orthoses if needed; an OT assessment at the hospital clinic and at home; a joint physiotherapy and OT school visit to discuss the diagnosis with teachers and provide advice as necessary for both school and home. Children were given equipment depending on their needs. This included: a writing wedge, hand putty, pen grips, cutlery grips and a laptop computer. Those with problems in their hands received an OT directed hand exercise programme. The intervention was conducted by a second paediatric physiotherapist (HB) and a paediatric occupational therapist (EJ). Both had access to baseline data in order to plan bespoke management.

Details of the specific structure of the intervention are listed below:

### Visit 1 (week 1)

A joint treatment session with the occupational therapist (EJ) and physiotherapist (HB). Children were given an individualised programme of exercise devised to target symptomatic joints and problem areas. Exercises were selected from the PhysioTools© manual. Information packs were supplied, and an orthotic appointment was arranged if deemed necessary.

### Visit 2 (week 2)

An OT review at home which included an assessment of activities of daily living, use of aids, sleep, and leisure pursuits.

### Visit 3 (week 4)

A school visit by both the physiotherapist and OT. This included the delivery of an education information pack and an assessment of difficulties in this environment. The child’s overall mobility around school, handwriting, physical education, and school attendance was discussed with teachers, the child was not provided with any treatment on this visit.

### Visits 4 and 5 (weeks 6 and 8)

These visits took place at the Jenny Lind Children’s Physiotherapy department at the Norfolk and Norwich University Hospital and were undertaken by the trial physiotherapist (HB). The assessments reviewed the individual exercise programme initiated on week 1 and adapted and progressed treatment as necessary.

### Main outcome measures

#### Outcome measures

The primary outcome measure was child reported pain (Wong-Baker scales). The secondary outcomes were parent-reported pain (100 mm VAS), physical function (CHAQ), HRQoL, coordination and grip strength. School attendance and requirement for health interventions were also recorded.

Primary and secondary outcome measures were assessed at 3 and 12 months after the baseline assessment. These outcomes were evaluated by trial physiotherapist (VE) who was blinded to the intervention group. Parents and children were specifically asked not to discuss their treatment with VE.

### Sample size

It was estimated that we would need to randomise 100 children to either of the two treatment arms to detect a 0.5 SD change in pain score between the intervention and the standard care groups (at a nominal one-sided significance of 0.05 and 80% power). This was estimated using the sample size commands in STATA statistical software [28]. We aimed to recruit 125 children to accommodate a 20% drop out rate anticipated from previous reported trials. No adjustment for multiple testing was made.

### Randomisation

The treatment groups were allocated randomly with minimisation used to achieve balance in group characteristics. The trial coordinator used a bespoke computer based method in the CTU remote from the research team. Three variables were utilised: age, gender and parent reported pain level.

### Statistical methods

As over 90% of the sample attended all assessments, an intention to treat complete case analysis was conducted. Comparisons were made between the two groups for data that related to the 12-month period starting at the date that the first assessment was completed.

Between group comparisons were carried out in primary and secondary outcome measures for the differences between baseline values and values at three months and 12 months and baseline by t-tests for unadjusted analysis and linear regression models for analysis adjusted for age.

An ancillary analysis was conducted to examine the change in primary and secondary outcome measures over time. Multilevel regression modelling was used, with fixed effects for intervention, time and treatment x time, and random effects for the intercepts at each time point. The analyses were conducted using the STATA statistical software version 12 [28].

### Ethical approval

The study was approved by the Norfolk Research ethics committee 09/H0310/80 on the 23rd of December 2009.

## Results

A total of 157 children were assessed for eligibility by the paediatric rheumatologist (KA). Of these 38 were not randomised: 11 did not meet the eligibility criteria and 27 declined consent.

**Fig. 1:**
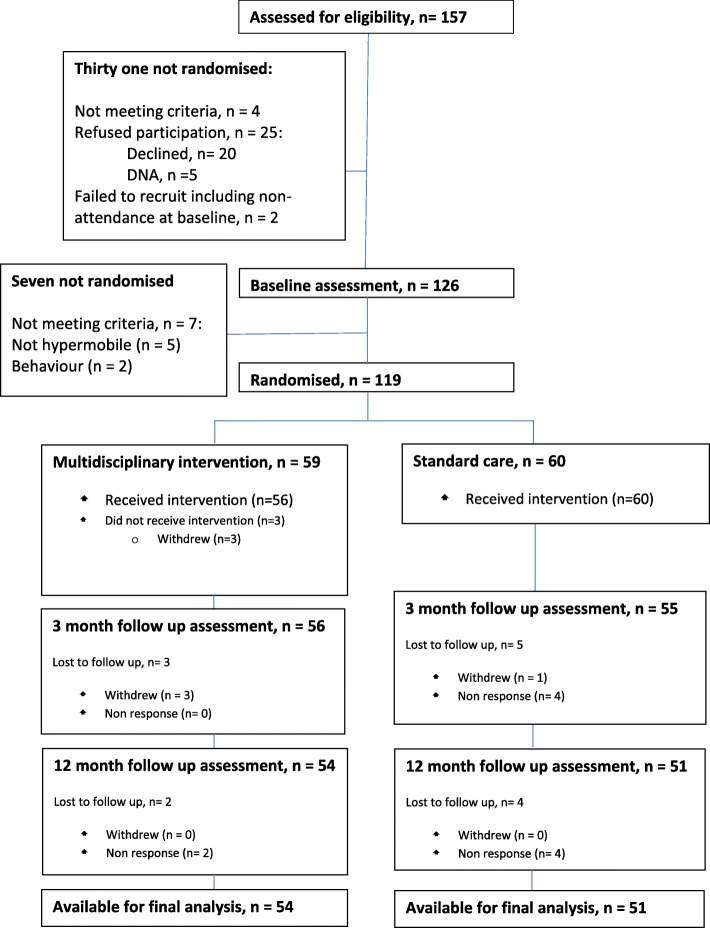
Participant flow through study

As illustrated in Fig. 1, 119 children were randomised, and 59 (50%) received the intervention. Of these, 111 attended 3 month follow up and 105 attended 12 month follow up. Of the 59 randomised to the intervention, 56 (93.3%) completed the multidisciplinary, multisite programme. All 56 attended 3 month follow up and 54 (91.5%) attended 12 month follow up. Of the 60 randomised to receive standard care, 55 (91.7%) were assessed at 3 months and 51 (85%) at 12 months. Overall the drop out was 6% at 3 months and a further 6% at 12 months.

### Characteristics of the sample

The children’s characteristics at their baseline assessment are shown in Table 2 and show no important differences between the two groups.

**Table 2:** Baseline demographic and clinical data, including hypermobility and symptom scores

Characteristics	Intervention (*n* = 59)	Standard care (*n* = 60)
Gender: male, *n* (%)	27.0 (45.8)	27.0 (45.0)
Age in years, mean (SD)	9.47 (3.18)	9.35 (3.20)
BMI, mean (SD)	18.9 (4.54)	18.0 (2.92)
Family history of JHS, *n* (%)	11 (18.6)	17.0 (28.3)
Child’s pain assessment, mean (SD)	2.19 (1.36)	2.53 (1.63)
Parent’s pain assessment, mean (SD)	33.8 (24.8)	40.6 (27.5)
Parent’s reported physical function, mean (SD)	0.84 (0.620)	0.860 (0.72)
Child reported HRQoL, mean (SD)	0.85 (0.100)	0.850 (0.12)
Coordination, mean centile (SD)	33.4 (26.7)	35.6 (30.1)
Grip strength (kilopascals), mean (SD)	57.0 (25.0)	59.4 (31.7)
Beighton, mean (SD)	5.80 (1.50)	5.70 (1.40)
Bulbena, mean (SD)	7.40 (1.60)	7.70 (1.30)
Number of painful joints, mean (SD)	1.80 (2.1)	2.10 (2.50)
Participation in sport (number of sessions per week), mean (SD)	2.60 (2.30)	2.60 (2.40)
Absence from school due to MSK problem (individual instances in last 3 months), mean (SD)	1.10 (3.30)	0.630 (2.10)
GP attendance for MSK problem (individual instances in last 3 months), mean (SD)	0.320 (0.800)	0.380 (0.880)
Hospital attendance due to MSK problem (individual instances in last 3 months), mean (SD)	1.10 (0.800)	1.47 (1.41)

### Outcomes at 3 and 12 months

Table 3 shows the between group differences in child pain as assessed by the Wong-Baker Faces scale between three months and baseline, and between 12 months and baseline. The level of pain improved in both groups at each of the follow up assessments; however, there was no significant difference in improvement between the two groups. A small improvement in child reported pain at 3 months was seen in the standard care group compared to the intervention group in the age adjusted data. Table 3 lists between group comparisons with baseline assessment for parent reported pain, parent reported physical function, HRQoL, coordination and grip strength. No change was observed in the child reported HRQoL score (CHU9D) between groups or in either group throughout the study period. Coordination improved more than expected for age related gains at 3 months, but was not generally sustained at a year. As might be expected grip strength improved with age. None of the differences between groups was significant.

**Table 3:** Differences in primary and secondary outcomes at 3 and 12 months

Factor	Standard care	Intervention	Unadjusted mean difference	*p*-value	Adjusted mean difference	*p*-value
*3 Months change from baseline (outcome – baseline)*						
Child’s pain assessment, mean (SD)	−1.33 (1.69)	−0.73 (1.55)	0.6 (− 0.02,1.22)	0.058	0.62 (0.04,1.21)	0.038
Parent’s pain assessment mean (SD)	−6.73 (26.26)	−3.27 (28.64)	3.46 (−6.88,13.8)	0.509	3.36 (−7.01,13.72)	0.522
Parent’s reported physical function, mean (SD)	−0.02 (0.38)	0.13 (0.52)	0.15 (−0.02,0.33)	0.088	0.15 (−0.02,0.32)	0.089
Child reported HRQoL, mean (SD)	0.00 (0.10)	0.01 (0.11)	0.01 (−0.03,0.05)	0.493	0.01 (−0.03,0.05)	0.492
Coordination, mean (SD)	7.57 (20.26)	6.21 (19.54)	−1.36 (−8.88,6.16)	0.721	−1.36 (−8.92,6.19)	0.721
Grip strength, mean (SD)	2.32 (12.03)	1.12 (13.87)	−1.2 (−6.18,3.78)	0.634	−1.21 (− 6.19,3.77)	0.632
*12 Months change from baseline (outcome – baseline)*						
Child’s pain assessment, mean (SD)	−1.58 (1.92)	−1.57 (1.53)	0.01 (−0.66,0.69)	0.967	0.06 (−0.6,0.71)	0.862
Parent’s pain assessment mean (SD)	−7.25 (29.50)	−6.81 (27.30)	0.44 (−10.55,11.43)	0.937	−0.08 (−10.94,10.78)	0.988
Parent-reported physical function, mean (SD)	−0.02 (0.38)	0.04 (0.55)	0.06 (−0.12,0.24)	0.522	0.06 (−0.13,0.24)	0.546
Child reported HRQoL, mean (SD)	−0.00 (0.12)	0.02 (0.09)	0.02 (−0.02,0.06)	0.244	0.03 (−0.02,0.07)	0.222
Coordination, mean (SD)	10.75 (19.46)	3.83 (20.75)	−6.92 (−14.76,0.92)	0.083	−6.87 (− 14.75,1.02)	0.087
Grip strength, mean (SD)	7.29 (16.05)	4.72 (17.16)	−2.58 (−9.05,3.89)	0.431	−2.59 (−9.1,3.92)	0.432

### Rate change in outcomes over time

The multilevel regression model is shown in Table 4 and shows a significant mean improvement in child and parent reported pain, coordination and grip strength over the 12 months of follow up across the intervention and the standard care group taken together. However, neither the intervention itself, nor any of the individual patient characteristics considered separately (age, gender, body mass index, and baseline degree of hypermobility) had a statistically significant impact on the rate of improvement over time. The self-reported baseline level of pain was a statistically significant determinant of 12-month outcome, with higher levels of baseline pain associated with more rapid mean reduction in pain score over time. A similar effect was seen for parent reported pain although it did not reach statistical significance.

**Table 4:** Multilevel modelling analysis

	Child’s pain assessment	Parent’s pain assessment	Parent-reported physical function	Child reported HRQoL	Coordination	Grip strength
Unconditional time effect	**−1.40 (−1–66, − 1.13)**	**−6.44 (− 12.0, −0.88)**	−0.0115 (− 0.121, 0.0979)	0.00992 (− 0.0142, 0.0340)	**5.51 (0.0373, 11.0)**	**5.56 (2.00, 9.12)**
Added univariate effects						
Intervention (vs. standard care)	−0.106 (− 0.630, 0.420)	0.056 (−11.4, 11.5)	−0.0639 (− 0.174, 0.302)	0.0146 (− 0.0311, 0.0600)	−5.74 (− 16.2, 7.74)	−2.49 (− 9.79, 4.81)
Age 9–12 (vs. 5–8))	0.475 (− 0.109, 1.06)	−6.99 (− 19.4, 5.45)	−0.101 (− 0.365, 0.164)	0.0155 (− 0.0364, 0.0674)	−3.55 (− 14.5, 7.37)	−0.642 (−7.41. 6.13)
Age 13–16 (vs. 5–8))	0.583 (− 0.135, 1.30)	− 12.99 (− 29.4, 4.18)	−0.0288 (− 0.316, 0.258)	0.0238 (− 0.0281, 0.0756)	3.65 (− 13.5, 20.8)	1.71 (−9.80, 13.2)
Female Sex (vs. Male)	0.426 (0.093,0.946)	−3.75 (− 15.5, 7.96)	−0.0702 (− 0.327, 0.187)	0.0304 (− 0.0435, 0.0900)	2.80 (− 7.78, 13.4)	−2.43 (− 9.77, 4.90)
BMI	0.062 (− 0.029, 0.153)	−0.33 (− 2.07, 1.41)	−0.00521(− 0.0220, 0.0324)	0.00306 (− 0.00345,0.00957)	−0.147 (− 1.73, 1.44)	0.806 (− 0.596, 2.21)
Beighton	0.013 (−-0.186, 0.212)	−0.48 (−4.88, 3.92	0.0154 (− 0.0762, 0.107)	−0.00305 (− 0.0188, 0.0127)	1.01 (−2.25, 4.27)	−0.0259 (− 2.67, 2.62)
Baseline child pain	**− 0.768 (−1.05, − 0.486)**	−3.35(− 7.31, 0.61)	0.0130 (− 0.0943, 0.0685)	0.0118 (− 0.00510, 0.0288)	1.07 (−2.41, 4.54)	−0.360 (− 2.46, 1.74)

Figures represent regression coefficients (95% CI) and indicate rate of change in the outcome variable per year per unit change in child and parent pain. BMI, and Beighton score, and per category for sex and age. Figures in bold are statistically significant at *p* < 0.05

## Discussion

This randomised, single centre parallel group trial compared a structured multidisciplinary, multisite intervention programme with standard care in children with symptomatic hypermobility. No added benefit was seen from this intervention. Both treatment groups showed improvements in child and parent reported pain scores, coordination and grip strength after one year follow up. The self-reported HRQoL (as assessed by CHU9D) did not change in either group through the period of follow up. Our HRQoL scores for both groups remained within the normal reference range for healthy children [29, 30], suggesting that while children with symptomatic hypermobility experience a degree of pain and motor impairment, these symptoms tend not to limit their overall wellbeing compared to their healthy peers.

Clinicians faced with diagnosing and planning treatment for children with hypermobility and pain currently have very sparse evidence on which to base their decisions. A systematic review [27] identified only three robust studies on the management of hypermobility [14, 31, 32], only two of which were conducted in the paediatric population [14, 32] and just one was a randomised comparative trial [14]. The non-randomised studies conducted in children were limited in their scope and targeted single joint-specific areas associated with hypermobility (knee proprioception and handwriting) [31, 32]. The single randomised trial focused on physiotherapy and compared targeted with a general programme of treatment. As in our own study, children showed an improvement in pain through follow up with no evidence to favour any particular management strategy. However, interpretation is limited by the small size (57 participants) and consequent lack of power, and low retention rate (just 56%). The influence of a fuller range of interventions that might be delivered by the multidisciplinary team has not been addressed.

The present study was designed to address the potential value of a multidisciplinary, multisite intervention package. Our intervention was developed through systematic literature review and clinical consensus, and adheres to the current British Society for Paediatric and Adolescent Rheumatology guidance. The intervention was designed for efficient delivery in a general hospital and community setting in a limited time.

This study has important limitations. We adopted a pragmatic approach to diagnosis, utilising a ‘real world’ clinical setting, and included children that an experienced paediatric rheumatologist diagnosed with symptomatic hypermobility. In taking this approach, we feel that the participants included reflect the broad range of the population referred to paediatric secondary and tertiary care and subsequently diagnosed with symptomatic hypermobility. Diagnostic criteria for JHS or Ehlers-Danlos hypermobility type (now termed hypermobility spectrum disorders and hypermobile EDS) [33, 34] have not been validated in the paediatric population. We applied Bulbena and Beighton scoring systems to confirm generalised hypermobility, to add face validity and to facilitate comparison with other published studies. The cut off values used conform to published literature, however population data on joint hypermobility scores across all age groups is still lacking. Higher cut off scores have been suggested but not validated [1, 34, 35].

All participants received at least ‘standard care’ as an ethical requirement and could not be blinded to the interventions, which require active engagement. Participants gave fully informed consent and were therefore aware of the interventions in the treatment arm, which may have influenced behaviour. The trial coordinator recorded the number of therapy attendances in the preceding 3 months at each follow up appointment in all participants to ensure that the assessing physiotherapist (VE) remained fully blinded. We are aware that in the standard care arm 36 children (60%) received at least one additional session of physiotherapy or occupational therapy and five received orthotics. Inevitably those randomised to the standard care group, irrespective of additional sessions, received a greater degree of attention than might have occurred outside an interventional trial.

We did not include direct measurement of child or parental anxiety in our study protocol. A systematic review of the literature conducted by our group has shown an association between anxiety and pain in children with hypermoblity [36]. It is possible that a reduction in anxiety due to clarification of the condition and education could have resulted in the improvements in pain seen across both groups.

A further limitation is the lack of validated outcome measures for symptomatic hypermobility in childhood. The pain measure used in the study assessed the intensity at the most prominent site and did not reflect the variable nature of pain or the variation in sites over the period assessed. The CHAQ is an assessment tool for children with juvenile idiopathic arthritis. It has not been validated for use in symptomatic hypermobility but has been used in a previous study [14]. It is conceivable that the outcome measures used may lack adequate sensitivity to detect a difference between the interventions. It is also possible that the sample size may have limited our ability to detect differences between the two intervention groups.

This study does not address whether hypermobile children report more pain compared to their non-hypermobile peers. In the multilevel model, there was no direct relationship between pain and degree of hypermobility and the degree of hypermobility was not related to level of symptom improvement or response to treatment. The data suggest that the relationship between the degree of joint mobility and a child’s experience of pain is not direct.

## Conclusions

We conclude that for children presenting with symptomatic hypermobility, standard care offered in routine practice in the UK is sufficient to lead to a sustained improvement in symptoms over a twelve-month period and that a more intensive multidisciplinary approach offers little further benefit. We recognise that symptomatic hypermobility is a heterogeneous condition and there may be subgroups that differ sufficiently to benefit from more intensive intervention [37, 38]. Nevertheless, our results suggest that for the majority the provision of education and positive interventions aimed at promoting healthy exercise and self-management leads to significant benefit without the need for more complex interventions. We believe our findings will help the development of prompt, simple and effective approaches to management, and will help diffuse beliefs about disability and negative consequences of this condition.

## Additional file

Additional file 1: Guidelines for Management of Joint Hypermobility Syndrome in Children and Young People. (PDF 458 kb)

## Abbreviations

CHAQ: Child Health Assessment Questionnaire (An outcome measure to assess physical functioning level)
CHU9D: Child Health Utility 9 dimensions (A validated tool to assess child reported health related quality of life/well-being)
CTU: Clinical trials unit
EDS: Ehlers-Danlos Syndrome (an umbrella term for a collection of hereditary connective tissue disorders)
HRQoL: Health related quality of life
JHS: Joint hypermobility syndrome
M-ABC2: Movement Assessment Battery for Children version 2 (a validated tool to assess childhood coordination)
NHS: National Health Service (UK governement healthcare provider)
UK: United Kingdom
VAS: Visual Analogue Scale (100 mm line to determine level of pain experienced 0 = no pain, 100 = maximum pain)
