# Supplementary material for: The effectiveness of a multidisciplinary intervention strategy for the treatment of symptomatic joint hypermobility in childhood: a randomised, single Centre parallel group trial (The Bendy Study)
Source: Pediatr Rheumatol Online J. 2019 Jan 8;17:2. doi: 10.1186/s12969-018-0298-x (PMC6325876; doi:10.1186/s12969-018-0298-x)
Supplement: Supplementary file 1 — Guidelines for Management of Joint Hypermobility Syndrome in Children and Young People. (PDF 458 kb) [file 12969_2018_298_MOESM1_ESM.pdf]

The British Society for Paediatric and Adolescent Rheumatology
Registered Charity # 1132967

Guidelines for Management of Joint Hypermobility Syndrome in Children and Young People.

A Guide for professionals managing young people with this condition

These guidelines have been compiled by the Allied Health Professionals Group of the British Society for Paediatric and Adolescent Rheumatology (BSPAR, 2012).
In order to cover the complexities of this condition some aspects of management have been divided into different professions, however there will be significant overlap as to who provides the intervention depending upon local teams.

Introduction
These guidelines are designed to help and support therapists working with children and young people with Joint Hypermobility and musculoskeletal pain.

Objectives

1. To facilitate the development of specialised, expert consensus opinioned and holistic management programmes for children and young people with Joint Hypermobility Syndrome (JHS), using a bio psychosocial model which acknowledges the global impact of the condition.
2. To optimise the standard of care of children and young people with JHS by empowering them, their carers and other health professionals through the provision of treatment, education, information and support.
3. To enable children, young people and their parents to be active participants in their self-management.
4. To ensure efficient, cost effective and evidence-based therapy management for children and young people with JHS.

These guidelines will be reviewed in 2015 by the BSPAR AHP group.

BSPAR is a Charitable Company registered in England and Wales. Company number 06978211 Registered office: 105 St Peter's Street St Albans Herts AL1 3EJ

Contents:

Assessment

a. Introduction

b. Subjective Assessment

I. Presenting Problems and Common Symptoms

II. Pain and Fatigue

III. Additional but rare symptomatology

i. Autonomic dysfunction

1. Gastrointestinal and urinary tract symptoms

2. Cardiovascular autonomic dysfunction

a. Orthostatic hypotension

b. Orthostatic intolerance

c. Postural orthostatic tachycardia syndrome (POTS)

ii. Other cardiac features

c. Objective Assessment

Management

I. Self-management

II. Pain Management

III. Physiotherapy

IV. Occupational therapy

V. Podiatry

VI. Clinical Psychology

VII. Pacing

VIII. School/Education

IX. Activity and Sport

X. Sleep management

XI. Weight management

XII. Mood and Motivation

XIII. Equipment

Concerning symptoms and behaviours

References

BSPAR is a Charitable Company registered in England and Wales. Company number 06978211 Registered office: 105 St Peter's Street St Albans Herts AL1 3EJ

Therapy Management Guidelines

Assessment

a. Introduction

Hypermobile joints are common in the general population and many individuals with hypermobility do not experience any significant difficulties. In fact in many situations (i.e. sportspeople, dancers etc.) hypermobility can have positive advantages. However some people experience difficulties and symptoms which are understood to be related to being hypermobile; commonly known as Joint Hypermobility syndrome (JHS) or Ehlers-Danlos syndrome Type III (Hypermobile EDS).

It is sometimes unhelpful for a young person to have the label of EDS III because the significant risks associated with the other forms of EDS can mistakenly be assumed to also apply to this group. For this reason, the preferred term to use is **Joint Hypermobility Syndrome** (JHS).

Assessing and managing this condition in children and young people requires specialist knowledge since for all the extra-articular symptoms including abdominal involvement, headaches, fatigue etc. there is a normal variance in the population. Care must be taken to ensure that appropriate importance is placed upon each symptom and the condition is not over-medicalised.

In general hypermobility should be regarded as a "***connective tissue advantage***" providing the child is strong and fit, difficulties occur mainly when the body has become weak and deconditioned.

Signs and Symptoms

There can be considerable variability in the severity of JHS, even within the same family. The main features are: joint hypermobility associated with muscle and joint pains and some level of fatigue. Easy bruising and clicking of joints are common symptoms which should not cause concern. Much less commonly young people may have problems with abdominal pain with or without some levels of bladder and bowel dysfunction and even rarer, postural orthostatic tachycardia syndrome (POTS), hernia, uterine or rectal prolapse and joint dislocation (though subluxation is slightly more common). There may also be an overlap with Developmental Co-ordination Disorder (DCD) and it may be difficult to differentiate between the symptomatic aspects of both without considering the criteria/definitions within the latest DSM assessment; however JHS is not life threatening and much benefit can be achieved from proactive and positive self-management with the goal being full participation in all activities.

Aims

JHS is often under recognised, not well understood and poorly managed. These guidelines are to provide further information for health professionals involved in the management of children and young people diagnosed with JHS. Patients have often been seen by a number of health professionals before a diagnosis is reached and families frequently report feeling that they haven't been listened to. Therefore, an important part of management is to listen to the family's concerns and deal with the expectations of both patients and parents.

The aims of treatment for JHS are to improve current symptoms, as well as avoiding future complications. Widespread and longstanding pain can result in a downward spiral of general physical

BSPAR is a Charitable Company registered in England and Wales. Company number 06978211 Registered office: 105 St Peter's Street St Albans Herts AL1 3EJ

deconditioning, pain and fatigue. A multi-systemic approach is needed to deal with the variable symptoms that can present. Each person with JHS is affected differently and so has varying management needs. It is important to work together with patients and families to develop an appropriate individual management plan. It is also helpful to establish realistic expectations along the way.

Management of JHS requires professionals, young people and their families to work together towards clearly defined and shared goals. The overall aim of any intervention is for the patient to gain a full understanding of hypermobility and the skills and knowledge to manage the condition themselves. The longer-term outlook for young people with JHS is very positive and with the correct management all young people should be able to participate in all activities they want to without on-going professional support. Most treatment centres would usually provide advice/orthotics/self-management programmes and would have limited resources for unlimited on-going treatment.

These guidelines outline the main issues that may need to be considered when developing an individualised management plan.

b. Subjective Assessment:

The subjective assessment of a child or young person with JHS should include a clear and comprehensive history covering the following areas:

- Presenting Problems
- Past Medical History
- Drug History
- Social and Family History
- Mental health and well being
- Ethnic and cultural concerns
- Benefits
- Participation in Activities of Daily Living (ADL) including self-care, leisure and sleep
- Impact on learning and participation in school activities
- Career planning / development
- What information has been given?
- Level of understanding of problems
- Occupational / Physiotherapy / Other Professionals

BSPAR is a Charitable Company registered in England and Wales. Company number 06978211 Registered office: 105 St Peter's Street St Albans Herts AL1 3EJ

I. Presenting problems and Common Symptoms:

- Joint and/or muscle aches and pains
    - Often occurring after activity or during the night more often lower limb than upper.
- Muscle and joint stiffness
    - Usually after exercise or increased activity, may occur for a few days after increased activity.
- Fatigue
    - Often associated with reduced walking distance and reduced exercise tolerance and /or difficulties attending fulltime education due to tiredness.
    - This symptom is commonly linked to level of deconditioning of the child.
    - Poor sleep will also impact upon fatigue levels.
- Fidgetiness, difficulty sitting still and poor organisation of movement etc
- Headaches
    - This is often related to muscle spasm of the Trapezius muscles.
- Poor sleep
- Easy bruising
    - This is benign and not of concern.
- Clicking joints
    - Joints can click spontaneously or be clicked deliberately, both are fine and can be performed safely many times a day. This only becomes a concern if it becomes habitual and obsessive impacting on QOL.
- Reduced co-ordination and balance
    - Poor proprioception leading to clumsiness and reduced balance.
    - Poor core stability leading to difficulties such as hand writing challenges and other issues with fine motor control.
    - Reduced fine motor control – especially involving small grip function.
    - If these symptoms do not improve with improvement of strength and gross motor skills and have significant impact upon ADL's and are significantly below what is expected for chronological age and intelligence level an assessment for a Developmental Co-ordination Disorder (DCD) may be advisable.
- Response to local anaesthetic
    - There is very little evidence to support this concern, however it has been reported that some people with JHS may have a reduced effect of a local anaesthetic.
- Abdominal pain
    - This is common in childhood generally and the incidence may be slightly higher in children who are hypermobile.
    - Constipation is a common cause of abdominal pain.

It is also important to remember that many of these symptoms have normal variants in the general population and care must be taken to ensure that there is a balance between medical investigation and effective symptom management.

BSPAR is a Charitable Company registered in England and Wales. Company number 06978211 Registered office: 105 St Peter's Street St Albans Herts AL1 3EJ

II. Pain and Fatigue

We usually understand pain to be a warning about damage to the body. When this is the case a reasonable response to the pain would be to withdraw or avoid activity.

However in JHS pain is an indication that the body is not strong enough to do the task and that it requires strengthening. This means that the young person will need to keep engaging in everyday activities and exercise, not do less.

Similarly fatigue is not necessarily an indication that more rest is required. Rather, it may signal that the body requires more fitness training, pacing of activities and better sleep hygiene.

It is also important to recognise that normal sleep patterns change throughout childhood into adolescence and then into adulthood. It is important to consider this when assessing fatigue levels and abnormal sleeping patterns.

Gradually pacing up activity levels and exercise is the most effective cure for fatigue during the day and often when there is a slump in energy levels a small snack and a brisk walk or other type of exercise will often rejuvenate the body in order to continue with the day's activities.

III. Additional but rare Symptomatology:

i. Autonomic dysfunction

Some children with JHS have been found to have an increased frequency of problems relating to the autonomic nervous system. Symptoms of autonomic dysfunction can occur at any age.

1. Gastrointestinal and urinary tract symptoms

Children and young people with JHS may have unspecific gastrointestinal symptoms, such as nausea, stomach ache, diarrhoea and constipation. Faecal and urinary incontinence can also be a feature of the condition. However these symptoms are also very common in children generally and may not be related to their hypermobility.

In very rare cases a disease called 'eosinophilic collitis' may be present. This is a condition that can cause constipation and difficulty maintaining a healthy weight and is often linked to food intolerances; however this condition can be well controlled with the guidance of a specialist gastroenterologist.

- Routine treatment of paediatric constipation is extremely effective with these symptoms.
- Advice about toilet habits can also be useful; such as having a stool to rest their feet on in front of the toilet to help optimise the position of the pelvis and enable effective bowel opening.
- If appropriate, arrange assessment of medical management of bowel symptoms through a paediatric gastroenterologist.
- Refer children or adolescents with significant urological problems for specialist assessment.

BSPAR is a Charitable Company registered in England and Wales. Company number 06978211 Registered office: 105 St Peter's Street St Albans Herts AL1 3EJ

2. Cardiovascular autonomic dysfunction

Patients with JHS may describe symptoms of cardiovascular autonomic dysfunction, which affects heart rate, blood pressure and blood flow. Again, these symptoms are very common throughout the general population especially during adolescence.

a. Orthostatic hypotension

A fall in blood pressure upon standing causes symptoms such as; venous pooling, dizziness, light headedness and syncope.

b. Orthostatic intolerance

Symptoms appear after periods of standing and can include; fatigue, light headedness, hyperventilation, sweating, paleness, anxiety, and acrocyanosis.

c. Postural orthostatic tachycardia syndrome (POTS)

POTS is characterised by a rapid rise in heart rate on standing (30 beats per minute above lying down heart rate). A common age of onset for POTS symptoms is during adolescence. Symptoms of POTS can include:

- Light-headedness and dizziness particularly on standing
- Fainting
- Heart palpitations or a racing heart beat
- Non -specific fatigue

Diagnosis of POTS is not easy and though it is often done by performing a tilt table test (or standing and lying heart rate if tilt table unavailable) this not validated in children and adolescents so the results of the tilt table test have to be interpreted carefully.

Treatment for POTS is available and usually involves simple measures such as increased fluid and salt intake. Exercise has been shown to be the most effective management for this aspect of JHS and medication should be used very carefully.

- Those with severe symptoms of cardiac autonomic dysfunction should be referred to a specialist clinic, such as a POTS clinic, or a Cardiologist with a specialist interest in syncope and arrhythmias.

ii. Other cardiac features

Increased aortic root size and mitral valve prolapse had been reported to be more common in patients with JHS, but current evidence suggests they are usually of little clinical consequence and is extremely rare. It is important to be careful not to alarm the child and their family and so referral for assessment of this should be carefully considered as increased anxiety about this condition is not helpful for the child.

- An echocardiogram may be recommended as a baseline assessment in extreme situations.

BSPAR is a Charitable Company registered in England and Wales. Company number 06978211 Registered office: 105 St Peter's Street St Albans Herts AL1 3EJ

Objective Assessment:

I. **General wellbeing**
   a. Patient VAS. A visual analogue scale for the perceived level of general wellbeing at the present time completed by the child if old enough.
   b. Parent VAS.

II. **Baseline measurements:**
   a. Height /weight /centiles / blood pressure
   b. Pain (VAS may be used)
   c. Fatigue (VAS may be used)

III. **Joint range of movement**
   a. Knowledge of where hypermobility is present is usually most effective in designing a management programme.
   b. Beighton Scale may be useful.

IV. **Muscle length**
   a. Awareness of muscles that move over 2 joints may become tight despite generalised Hypermobility.

V. **Muscle strength**
   a. A basic assessment of muscle strength is vital. Often scoring using the Kendal Scale (0-10) provided a score that easily monitors change over time.
   b. Specific muscles should always be assessed including:
      i. Inner Range Quads (Straight Leg Raise without a quadriceps lag)
      ii. Hip abductors – specifically Gluteus Medius
      iii. Hip Extensors – specifically Gluteus Maximus
      iv. Plantar Flexors
      v. Core central Stability

VI. **Posture and Gait**
   a. Both should be assessed especially with the view to prescribe orthotics.

VII. **Stamina**
   a. 6 min walk test may be useful.
   b. Subjective reporting of distance and time walked is also effective.

VIII. **Function**
   a. The CHAQ may often demonstrate how significantly independent function is affected by this condition. (CHAQ is not formally validated for JHS).
   b. An objective assessment of occupational performance including self-care, leisure and activities at school such as handwriting, dextrous manipulation, activity organisation, dressing etc.

BSPAR is a Charitable Company registered in England and Wales. Company number 06978211 Registered office: 105 St Peter's Street St Albans Herts AL1 3EJ

Management of JHS

The Multi-Disciplinary Team:

As some young people with JHS can present with a wide variety of difficulties, they may need to be seen by a number of different professionals who must ensure that they communicate with each other in order to provide a cohesive management programme. A thorough MDT assessment will help determine the most appropriate individualised treatment plan.

I. Self management

It is helpful for families to understand JHS and that this is a non life threatening condition and in many instances hypermobility can be advantageous.

The aim for young people and their families is for them to develop the skills and knowledge in order to manage the symptoms and participate in all activities.

For the best chance of success parents and adolescents should be fully involved in the treatment plan and age appropriate language must be used with children.

People with JHS do benefit from maintaining a healthy everyday life. A well balanced diet, regular gentle exercise, full participation in school and activities as well as quality sleep all play their part.

Children and adolescents may benefit from advice on best technique for carrying out tasks of daily living, to avoid placing unnecessary strain on their joints and minimising pain. Often encouragement is needed to include appropriate exercise into their daily routines. It is well recognised that significant periods of inactivity greatly exacerbate symptoms of JHS.

A good understanding of pacing activities needs to be developed by children and adolescents with JHS in order to avoiding the 'boom and bust' cycle (over exertion leading to pain and fatigue). As children and adolescents grow up, gaining an understanding themselves about JHS will help them develop the motivation needed to manage their condition and develop a healthy lifestyle incorporating regular exercise.

II. Pain Management / Education

Recognition and management of chronic pain is an important part of management for children with JHS. Pain can affect concentration, memory, mood and sleeping. If appropriate a referral to a unit that is confident with the management of chronic pain in children may be helpful as chronic pain is most effectively managed with a combination of physical and psychological techniques.

Pain medications are often ineffective and the side effects should be carefully considered. Pain management interventions can be provided by physiotherapists, occupational therapists and psychologists depending upon the local service provision.

The aim of pain management is to increase quality of life. Therefore patient-led goals will be an important part of boosting motivation to engage in the self-management programme. Fear of the unknown can be a big source of anxiety for both the young person and their family, so education and addressing the personal beliefs can be helpful in reducing such fears. Cognitive restructuring can help to recognise unhelpful behaviours, such as excessive rest, and establish new patterns of reasoning. It is important to identify any catastrophising and avoidance behaviours and to recognise their role in the fear cycle.

BSPAR is a Charitable Company registered in England and Wales. Company number 06978211 Registered office: 105 St Peter's Street St Albans Herts AL1 3EJ

For children and young people with JHS it is helpful to consider their relationships with others. Parents can be hugely disempowered if their child is experiencing pain. It is therefore important to help parents to develop strategies that they can use to ensure they promote positive rather than unhelpful patterns of behaviour for their child.

It is helpful to establish realistic expectations. There will be times of increased pain, for example after a lot of sport or injury and a management plan for dealing with these times should be discussed. It is really important to ensure that at these times rest is not promoted for long periods of time.

In general, medical approaches to pain management are not thought to be particularly beneficial for JHS, with patients reporting that analgesics have limited effect and there is the disadvantage of side effects that come with certain medicines.

COMFORT TOOL BOX: it is useful for the child and family to develop their own collection of activities and interventions that can be used to reduce the pain. These can be items that facilitate distraction as well as positive coping statements, relaxation scripts, aromatherapy oils, exercises, physical activities comforting objects and pictures etc. This may be a real box full of these things or a virtual box that have been created between the young person and their family.

III. Physiotherapy

A lifelong commitment to exercise is needed for all patients with JHS. Physiotherapy can help to promote this from a young age using specific exercises and encouraging activities that can be incorporated into daily life and, most importantly, be enjoyed!

The plan:

- Restore and maintain full muscle strength and function throughout the full range of movement.
- Restore effective and efficient movement patterns.
- Improve general fitness.
- Restore normal range of movement, including into hypermobile range.
- Provide education, reassurance, advice, pain management and develop problem solving.

The goal:

- Self-management.

Patients with JHS often experience pain. Left unresolved, this may lead to fear of movement and decreased activity, resulting in muscle deconditioning. Core and specific muscular strength is vital for patients with JHS to help minimise pain, maintain good posture and reduce joint instability. Activities to maintain strength and stamina are vital for managing this condition.

However, patients with JHS tend to have a poor tolerance of static, repetitive or excessive activity, often resulting in pain and fatigue the following day. This is due to having specific muscle imbalances resulting in “delayed onset muscle pain” (DOMS) and joint discomfort. This can cause worries about aggravating symptoms and result in a reluctance to exercise. However DOMS is a completely normal response to an increased level of exercise and is not an indication of damage.

BSPAR is a Charitable Company registered in England and Wales. Company number 06978211 Registered office: 105 St Peter’s Street St Albans Herts AL1 3EJ

Patients with JHS may have reduced proprioception, making it harder for them to carry out exercises correctly on their own. Incorrect positioning may result in exercises being ineffectual or damaging. Children with JHS are often hypersensitive and become more aware of the pain sensations and so this should be taken into consideration when using massage or manual treatment techniques.

The physiotherapy will be most effective if targeted to the specific areas of difficulty the young person is experiencing. This may be a specific muscle weakness or a specific function that is difficult. Often a specific exercise programme will ensure that the muscles can protect the hypermobile joints enabling full inclusion in all activities. In order to engage children and young people, physical therapy should aim to be specific, effective, time efficient and if possible FUN! Consider the patient's goals and what will be achievable.

- **Normal activities**

  Encourage normal activities and a return to sport, providing individual advice on specific elements of activities as appropriate.

- **Specific exercise programme**

  The most effective exercise programme for the management of hypermobility will be a progressive resisted exercise programme that targets the specific muscles that are weak and that are required to control the joints into their hypermobile range. If the muscles are completely effective then posture and function will improve. The use of open chained exercises, non-weight-bearing which build on the principal of high repetitions and low weights has been shown to be very effective. These exercises can be started at a very early age.

- **Advise on postural alignment**

  Good postural alignment protects supporting structures against injury, enables muscles to function most efficiently and provides optimum positions for thoracic and abdominal organs.

- **Core Strengthening**

  Exercises to build core strength, muscular strength and endurance improve stability, balance and coordination. Incorporate functional and everyday activities. Try balance boards, wobble cushions and gymnastic balls as well as specific exercises.

- **Stretching**

  Stretching can be used to maintain muscle length, joint range and to stretch out old injuries or muscle spasms. Stretching should not be to increase an already hypermobile range.

- **Joint Subluxations**

  Repeated self-subluxation of joints should be avoided, however resting in "unusual" postures i.e. "W" sitting is fine as this is in their normal range of movement.

- **Goal setting**

  'Realistic' goal setting (both short and long term) can help patients in making progress.

- **Proprioception training**

  Exercises to enhance proprioception can include the use of mirrors, bio feedback and balance boards may also be effective as well as the strengthening exercises. Programmes to enhance proprioception have been proven to show good results.

BSPAR is a Charitable Company registered in England and Wales. Company number 06978211 Registered office: 105 St Peter's Street St Albans Herts AL1 3EJ

- **Sport specific assessment**

  Assessment of swimming and other sports techniques may be helpful to enable to young person to participate fully.

IV. Occupational Therapy

Occupational therapists can assess a child's performance in a range of daily activities. This may be done in a clinic setting, in the home or school environment. Assessment will be holistic and will consider the child's skills, their motivation and their daily routine, as well as their environment. Comprehensive assessment will allow the OT to identify barriers to occupational performance and the design the most appropriate intervention. This may include building skills; suggesting strategies, educating the young person and others in order to enable independence in daily activities.

Those with JHS have greater difficulty building up muscle strength and endurance and most will be familiar with the 'boom and bust' cycle associated with overuse. Pain will often after activity which may lead to children avoiding particular activities which may have a marked impact on their self-confidence and self esteem

Pacing

Pacing which refers to a systematic graded approach to participation in activity, along with energy conservation is vital so that young people learn to use their energy for the activities they need and want to do. An activity diary will shed light on the current level and pattern of activity. From this, an activity baseline which is easily manageable with minimal effort can be set. Over time the level of activity should be increased until they have enough stamina and coping strategies to manage all activities.

Daily occupations

Assessment of participation in daily activities will be important. Interventions are likely to include both physical and psycho social strategies. The chief focus should be on building skills and resilience. Rarely would assistive devices (such as splints) as a compensatory approach prove beneficial in the longer term. Simple adaptations such as pen grips may be very effective and there are a large variety of different styles and shapes of pen and pencil which can be found that will enable writing to be more efficient.

Home, school and leisure should all be considered and close liaison with the school would be important to ensure the most appropriate support is provided in a school environment.

Sleep and relaxation

For those experiencing difficulties with sleep, sleep hygiene advice may be useful. A careful assessment of sleep patterns will be helpful in targeting other interventions such as distraction, stress management and relaxation techniques.

Pain management

The OT profession is founded on the principal that there is a direct link between activity, health and well-being, they are therefore well placed to also help the young person explore the inter relation between physical symptoms, feelings/emotions and activity and offer appropriate advice or intervention.

BSPAR is a Charitable Company registered in England and Wales. Company number 06978211 Registered office: 105 St Peter's Street St Albans Herts AL1 3EJ

Self confidence

In some instances pain and difficulty engaging in a full range of every -day activities may have impacted on a child/young person's self-esteem and motivation level. OT's will promote meaningful activities aimed at increasing this to ensure satisfactory participation in daily life.

Splinting

Splinting is generally avoided due to the risk of deconditioning, but occasionally for the hypermobile MCP of the writing hand a splint is useful to prevent the hyperextension therefore aiding writing, reducing pain and fatigue.

V. Podiatry

Podiatrists provide advice on suitable foot wear. Slip-on shoes should be avoided and supportive boots/ shoes that are purchased from appropriate shoe shops may be considered for children with weak ankles. Often boots with laces or high-top trainers are appropriate and comfortable footwear.

Orthotics may be prescribed for those with flat feet where the positioning and function of the feet may contribute to symptoms.

VI. Clinical Psychology

Clinical psychology may be required to ensure that JHS does not prevent the young person fully participating in life.

Psychology input may focus upon pain management skills and techniques or on thoughts, feelings and behaviours in response to symptoms. Cognitive behaviour therapy (CBT) may be useful in modifying unhelpful beliefs and behaviours, though many other approaches may also be utilised.

If the young person is experiencing low mood and/or anxiety these may negatively impact upon their symptoms and their ability to self-manage. A clinical psychologist would be able to work with the young person and their family in order to help modify these feelings so that they are able to regain control of the symptoms and their life.

VII. Pacing

It is important for all young people with JHS to balance periods or activity and rest in order to maximise their ability to achieve physically, emotionally and intellectually. A good understanding of pacing can help avoid the 'boom and bust' cycle (over exertion leading to pain and fatigue and consequent under-activity). It is also important to recognise that initially the balance between rest and exercise/activity may have more time spent resting but the goal will be to increase the activity levels and reduce the rest until the balance is tipped back to normal activity levels during the day and the majority of rest gained at night. Once a young person has recovered to this level, pacing can be reduced and in many cases stopped altogether as the young person is able to engage in everyday activities, including full time school, without an increase in symptoms.

VIII. School/Education

Children and young people with JHS need to be able to engage in regular education just like any other child. From time to time they may need extra support to achieve their best in school, and some minor modifications may be appropriate.

BSPAR is a Charitable Company registered in England and Wales. Company number 06978211 Registered office: 105 St Peter's Street St Albans Herts AL1 3EJ

Guidance from health professionals working with the individual may help school staff understand the impact the child's condition. Most pupils do not like to feel 'different'. It is important for emotional well-being to enable pupils with JHS to be included as much as possible in school activities, but with appropriate modifications.

- An OT assessment within school may be beneficial.
- Suitable sporting activities should be encouraged, with advice on the suitability of high impact activities and allowances made for reduced stamina.
- Appropriate seating may help with posture and comfort in the classroom and movement can help avoid discomfort and pain. It is often helpful to ensure that the child does not stay still for extended periods of time. For example asking the child to do small tasks that require getting up and walking about may prevent discomfort without disrupting the class.
- Pupils with JHS may need extra time to move between classes and travel around the school. For pupils with increased pain leaving classes early maybe helpful.
- After an OT assessment it may be appropriate to recommend that extra time is given during exams and that the use of typing the exam may help as well.
- Urinary urgency and bowel problems may be part of JHS so access to toilets should not be restricted.
- Storage may be helpful for books and bags so they don't have to be carried around school all day.
- A Hypermobility guide for schools booklet is available from the Hypermobility Syndrome Association (HMSA).

IX. Activity and Sport

Physical activity should be actively encouraged for all young people with JHS. However it is extremely important that the child is fit enough to engage in the sport they wish to do. If this is not the case, young people may need to gradually build up their strength and stamina before engaging in the new activity. Often the physiotherapy programme can be used to ensure that every muscle can do its 'job' effectively. There are some sports that will need more preparation for than others such as contact sports.

Care, however needs to be taken when considering trampolining and bouncy castles as these activities put significant stress on the joints and often involve several children at a time which can increase the risk of injury.

With the correct preparation any sport can be considered. However in childhood it is often better to enjoy a variety of sports throughout the week rather than focus on just one.

X. Sleep management

Children and young people with JHS often describe poor and disturbed sleep which may be a result of pain or poor pacing skills resulting in too much rest during the day making it difficult to get to sleep at night. Parents can help to ensure routine and good sleep hygiene. Relaxation techniques may also help promote sleep. Additional professional advice is available if needed.

BSPAR is a Charitable Company registered in England and Wales. Company number 06978211 Registered office: 105 St Peter's Street St Albans Herts AL1 3EJ

XI. Weight Management

Maintaining optimum body weight is important. Extra weight can put significant strain on the joints and increase the symptoms of pain and fatigue. Advice may be provided about healthy diets and this may also help in the management of constipation.

It is equally important not to become underweight as this can make maintaining adequate muscle strength difficult. In this instance, it may not be safe to engage in an intensive exercise programme until the young person is at a healthy weight. Weight should be monitored during any intervention.

XII. Mood and Motivation

Understandably, experiencing any of the symptoms described above can have a negative impact on mood, which can reduce the person's ability to engage in everyday activities and the recommended management programme. Lack of activity and participation, in turn can further exacerbate the symptoms. Low mood and anxiety are commonly experienced by people stuck in such a negative cycle and they may start to avoid activities such as a result of a fear of causing more pain and /or fatigue. A thorough MDT assessment will help to identify individuals/families that may benefit from further support in managing emotional distress associated with chronic symptoms.

XIII. Equipment

The general principal is that special equipment is not required. There should never be the provision of a wheelchair for a young person with JHS as this is very damaging to the requirement of maintaining full strength and function. Crutches can be equally unhelpful and should also be avoided in the management of JHS.

The general philosophy applies to the provision of equipment in that the most effective solution to a physical challenge is to find a way to get stronger and fitter so that it can be done with minimum adaptations.

Concerning Symptoms

Unfortunately for some families the philosophy of self-management and maintaining strength and full independent function is very challenging and their fear of the pain and symptoms may be such that they find following the advice of the Professionals very difficult. These families may need more time and interventions in order to help them understand the condition and the real meaning of the pain messages and it may be that for these families the psychological input, in order to help them modify their beliefs, may be the first treatment required. At this time a Multidisciplinary assessment including a level 3 Child Protection trained Paediatric Rheumatologist or Paediatrician and a Psychologist would be appropriate..

BSPAR is a Charitable Company registered in England and Wales. Company number 06978211 Registered office: 105 St Peter's Street St Albans Herts AL1 3EJ

Peer Support

Therapists should also ensure that families are aware of organisations, which may be able to offer emotional, financial and practical support. A number of organisations that provide such help are listed below. Resources include handbooks, newsletters, and family holidays.

Hypermobility Association HMSA	Phone: 0845 345 4465<br>Email:<br>Web: http://www.hypermobility.org	**The Hypermobility Syndrome Association**<br>49 Orchard Crescent<br>Oreston<br>Plymouth<br>PL9 7N

Key / Audit points:

- **Self management of the symptoms is vital for the long-term effective management of JHS in children and young people. This may include a specific exercise regime and active participation in non-pharmacological pain management techniques to ensure full participation in all activities.**
- **Pain management and education is an important role of any Allied Health Professional involved in the care of a child or young person with JHS.**
- **Liaison with school is an important role of the AHP in the care of young people with JHS.**

References

The following texts were used as reference and may be helpful for further reading:

Brad T. Tinkle, MD, PHD (2008). Issues and Management of Joint Hypermobility. A Guide for the Ehlers-Danlos Hypermobility Type and the Hypermobility Syndrome. Left Paw Press 2008

Rosemary Keer & Rodney Grahame (2003). Hypermobility Syndrome: Recognition and Management for Physiotherapists. Butterworth Heinemann 2003.

Acknowledgements:

Multi-disciplinary team members of the British Society for Paediatric and Adolescent Rheumatology.
Produced by: Sue Maillard, Catherine Stott and Sue Kemp
Issue number 1.1, Jan 2013
Ratified by BSPAR Executive Committee: 20.6.13
To be reviewed 2015

BSPAR is a Charitable Company registered in England and Wales. Company number 06978211 Registered office: 105 St Peter's Street St Albans Herts AL1 3EJ
